# Challenges and opportunities in the management of type 2 diabetes in patients with lower extremity peripheral artery disease: a tailored diagnosis and treatment review

**DOI:** 10.1186/s12933-024-02325-9

**Published:** 2024-06-26

**Authors:** Guillaume Mahé, Victor Aboyans, Emmanuel Cosson, Kamel Mohammedi, Gabrielle Sarlon-Bartoli, Damien Lanéelle, Tristan Mirault, Patrice Darmon

**Affiliations:** 1 Vascular Medicine Unit, University Hospital of Rennes, Rennes, France; 2 Clinical Investigation Center, CIC 1414, INSERM, Rennes, France; 3 M2S– EA 7470, University of Rennes, Rennes, France; 4 Department of Cardiology, Dupuytren-2 University Hospital, Limoges, France; 5 EpiMaCT, Inserm 1094 & IRD 270, Limoges University, Limoges, France; 6 AP-HP, Avicenne Hospital, Department of Endocrinology-Diabetology-Nutrition, CRNH-IdF, CINFO, Paris 13 University, Sorbonne Paris Cité, Bobigny, France; 7 Nutritional Epidemiology Research Unit, UMR U557 INSERM/U11125 INRAE/CNAM, Paris 13 University, Sorbonne Paris Cité, Bobigny, France; 8 Department of Endocrinology, Diabetes, and Nutrition, University Hospital of Bordeaux, Pessac, France; 9 INSERM, BMC, U1034, University of Bordeaux, Pessac, France; 10 Vascular Medicine and Hypertension Department, La Timone University Hospital of Marseille, Marseille, France; 11 Centre for Nutrition and Cardiovascular Disease (C2VN), Faculty of Medicine, Aix-Marseille University, Marseille, France; 12 Department of Vascular Medicine, Caen Normandy University Hospital, Caen, France; 13 COMETE, INSERM, GIP Cyceron, University of Caen Normandy, Caen, France; 14 Vascular Medicine Department, Hôpital Européen Georges-Pompidou, Assistance Publique Hôpitaux de Paris, Université Paris Cité, Paris, France; 15 Institut des Sciences Cardiovasculaires, Paris Cardiovascular Research Center, INSERM U970, Université Paris Cité, Paris, France; 16 Department of Endocrinology, Metabolic Diseases, and Nutrition, Assistance Publique-Hôpitaux de Marseille (AP-HM), University Hospital Conception, Marseille, France

**Keywords:** Type 2 diabetes, Peripheral artery disease, Guidelines, Management, Glycemic control

## Abstract

Lower extremity peripheral artery disease (PAD) often results from atherosclerosis, and is highly prevalent in patients with type 2 diabetes mellitus (T2DM). Individuals with T2DM exhibit a more severe manifestation and a more distal distribution of PAD compared to those without diabetes, adding complexity to the therapeutic management of PAD in this particular patient population. Indeed, the management of PAD in patients with T2DM requires a multidisciplinary and individualized approach that addresses both the systemic effects of diabetes and the specific vascular complications of PAD. Hence, cardiovascular prevention is of the utmost importance in patients with T2DM and PAD, and encompasses smoking cessation, a healthy diet, structured exercise, careful foot monitoring, and adherence to routine preventive treatments such as statins, antiplatelet agents, and angiotensin-converting enzyme inhibitors or angiotensin receptor blockers. It is also recommended to incorporate glucagon-like peptide-1 receptor agonists (GLP-1RA) and sodium-glucose cotransporter-2 inhibitors (SGLT2i) in the medical management of patients with T2DM and PAD, due to their demonstrated cardiovascular benefits. However, the specific impact of these novel glucose-lowering agents for individuals with PAD remains obscured within the background of cardiovascular outcome trials (CVOTs). In this review article, we distil evidence, through a comprehensive literature search of CVOTs and clinical guidelines, to offer key directions for the optimal medical management of individuals with T2DM and lower extremity PAD in the era of GLP-1RA and SGLT2i.

**Supplementary Information:**

The online version contains supplementary material available at 10.1186/s12933-024-02325-9.

## Introduction

Type 2 diabetes mellitus (T2DM) stands out as a potent risk factor for lower extremity peripheral artery disease (PAD), with individuals with diabetes facing a two-fold higher risk of developing PAD compared to those without diabetes [1]. In comparison to individuals without diabetes, those with T2DM exhibit a more severe manifestation, partly related to concomitant neuropathy, as well as a more distal distribution of PAD, increasing the risk of complications [2, 3]. Patients with concomitant T2DM and PAD are at a high risk of cardiovascular events, including lower-limb events [2, 4]. Moreover, patients with PAD and T2DM are 2–10× more likely than non-diabetic patients to undergo an amputation [2, 5]. Indeed, approximately 70% of cases undergoing lower-extremity amputation in the United States are attributed to diabetes [6]. Diabetes-related amputations lead to profound functional disability, placing an immense economic burden on both patients and health systems [7]. Globally, 113 million individuals aged 40 years and older are living with PAD [8], of whom about 20–30% present with concomitant T2DM [9, 10].

Although T2DM may alter the clinical presentations of PAD, the diagnosis of PAD is often straightforward through non-invasive measures, such as the resting ankle-brachial index (ABI) and the toe-brachial index (TBI) [11, 12]. Conversely, the medical management of PAD, particularly in patients with T2DM, raises considerable challenges. While the focus has primarily been on managing major cardiac events in patients with T2DM, the care for PAD has experienced a more gradual progression in terms of evidence-based therapies [12, 13]. Notably, the benefits of novel glucose-lowering agents, such as glucagon-like peptide-1 receptor agonists (GLP-1RA) and sodium-glucose cotransporter-2 inhibitors (SGLT2i), for individuals with PAD remain overshadowed within the background of cardiovascular outcome trials (CVOTs). Existing data are often derived from post-hoc analyses of PAD subgroups, and offer, at best, low-grade evidence [14–18]. In addition, the types of medical and surgical specialties involved in PAD care vary significantly across the globe, and even within individual countries, leading to a heterogeneous and non-standardized patient pathway. The lack of standardized treatment protocols and organizational structures further contributes to the complexity of the management of patients with T2DM and PAD [19]. Therefore, pending the results of future clinical trials, multidisciplinary teams, including endocrinologists/diabetologists, vascular specialists, and primary care practitioners, have the opportunity to optimize the benefits gained from the existing treatment armamentarium in patients with both T2DM and PAD. In this review article, we distil evidence, through a comprehensive search of the literature and clinical guidelines, to offer key directions for the optimal medical management of patients with T2DM and lower extremity PAD in the era of GLP-1RA and SGLT2i.

## Search strategy

Two industry-independent systematic literature searches were performed on MEDLINE (PubMed). The first search, which was conducted in October 2023, aimed to identify position statements, expert consensuses, and societal guidelines on the treatment of PAD in patients with T2DM. The first search used the keywords: (“diabetes”) AND (“PAD” OR “peripheral artery disease” OR “peripheral arterial disease” OR “lower extremity arterial disease” OR “lower extremity artery disease” OR “LEAD”). In total, we found 15 clinical practice guidelines related to the therapeutic management of patients with T2DM and PAD (Table 1). The second search, which was conducted in November 2023, focused on identifying randomized controlled trials (RCTs) and CVOTs evaluating newer glucose-lowering agents for PAD, mainly GLP-1RA and SGLT2i. The second search included the keywords: (“PAD” OR “peripheral artery disease” OR “peripheral arterial disease” OR “amputation*” OR “foot ulcer”) AND (“glucagon-like peptide-1” OR “glucagon-like peptide 1” OR “GLP-1” OR “GLP1” OR “GLP-1RA” OR “SGLT2” OR “SGLT2i” OR “sodium-glucose cotransporter 2” OR “sodium-glucose cotransporter-2” OR “liraglutide” OR “semaglutide” OR “dulaglutide” OR “exenatide” OR “lixisenatide” OR “efpeglenatide” OR “canagliflozin” OR “empagliflozin” OR “dapagliflozin” OR “ertugliflozin” OR “sotagliflozin”). In total, we found 15 CVOTs or RCTs evaluating GLP-1RA and SGLT2i for PAD (Table 2; Fig. S1).

**Table 1:** Recommendations on the treatment of peripheral artery disease (PAD) in patients with type 2 diabetes mellitus (T2DM), as outlined by different clinical practice guidelines

Recommendation	ACC/AHA [11]	ADA [20–22]	ADFDG working group [23]	CCS [24]	CDS [25]	ESC/ESVS [12, 13, 26–28]	GDS [29, 30]	SFMV/SCVE [31]
Smoking cessation	X	X	X	X		X	X	X
Structured exercise therapy, in particular supervised exercise training	X	X		X		X	X	X
Mediterranean diet		X				X		X
Lipid-lowering agents: Statins, with additional lipid-lowering therapy with ezetimibe or a PCSK9 inhibitor if target lipid levels not achieved	X	X	X	X	X	X	X	X
Tight glycemic control (HbA1c < 7.0%)	X	X		X		X		X
Antihypertensive agents: ACEis or ARBs	X	X		X		X	X	X
Long-term antiplatelet therapy with clopidogrel or aspirin in patients with symptomatic PAD	X	X	X	X	X	X	X	X
Combination of rivaroxaban (2.5 mg twice daily) and aspirin (up to 100 mg once daily) in patients with symptomatic PAD at a low risk of bleeding		X		X		X	X	X
Revascularization in case of a lifestyle-limiting claudication or a chronic limb-threatening ischemia	X	X	X	X	X	X	X	X
Revascularization decisions based on individual factors (i.e., length, anatomic location, and extent of obstructive disease, availability of autogenous vein, patient comorbidities, local expertise)	X	X	X	X	X	X	X	X

ACC, American College of Cardiology; ACEi, angiotensin-converting enzyme inhibitor; ADA, American Diabetes Association; ADFDG, Australian Diabetes-related Foot Disease Guidelines; AHA, American Heart Association; ARB, angiotensin-receptor blocker; CCS, Canadian Cardiovascular Society; CDS, Chinese Diabetes Society; ESC, European Society of Cardiology; ESVS, European Society for Vascular Surgery; GDS, German Diabetes Society; HbA1c, glycated hemoglobin; PCSK9, proprotein convertase subtilisin/kexin type 9; SCVE, French Society for Vascular and Endovascular Surgery; SFMV, French Society of Vascular Medicine

**Table 2:** Analyses of cardiovascular outcome trials (CVOTs) assessing sodium-glucose cotransporter-2 inhibitors (SGLT2i) and glucagon-like peptide-1 receptor agonists (GLP-1RA) for peripheral artery disease (PAD)

Trial(s)	References	Study description	Follow-up	Main findings
*SGLT2i*				
CANVAS & CANVAS-Renal (canagliflozin)	Matthews et al. [32]	Post-hoc analysis of 2 double-blind, randomized trials involving 10,142 patients with T2DM and a history or a high risk of CV disease who received canagliflozin (100 or 300 mg/day orally) vs. matching placebo	Mean of 3.6 years (5.7 in CANVAS and 2.1 years in CANVAS-R)	Rates of amputations were 6.30 and 3.37 events per 1000 participant-years with canagliflozin vs. placebo (HR 1.97; 95% CI 1.41–2.75) Risk factors for amputation included: history of amputation (HR 16.27; 95% CI 10.65–24.63), history of peripheral vascular disease (HR 2.77; 95% CI 1.93–3.96), and history of neuropathy (HR 1.86; 95% CI 1.35–2.56)
CANVAS & CANVAS-Renal & CREDENCE (canagliflozin)	Yi et al. [14]	Post-hoc analysis of 3 double-blind, randomized trials in 14,543 patients with T2DM, of which 3514 had CKD without PAD and 1156 had CKD and PAD, who received canagliflozin (100 or 300 mg/day orally) vs. matching placebo	Median of 2.5 years	In those with CKD and PAD, canagliflozin reduced risk of MACE (HR 0.62; 95% CI 0.47–0.83), composite of HHF or CV death (HR 0.62; 95% CI 0.46–0.82), and composite of ESKD or doubling of serum creatinine (HR 0.51; 95% CI 0.33–0.79), with no heterogeneity of effect with canagliflozin between patients with and without PAD (p_interaction_ > 0.20) No increase in serious AEs or lower-limb amputations was observed with canagliflozin in patients with CKD, regardless of PAD status (*p* = 0.33)
CANVAS & CANVAS-Renal & CREDENCE (canagliflozin)	Barraclough et al. [33]	Post-hoc analysis of 3 double-blind, randomized trials in 14,543 patients with T2DM, of whom 3159 (21.7%) had PAD at baseline, who received canagliflozin vs. matching placebo	Median of 2.5 years	In patients with PAD, canagliflozin reduced MACE (HR 0.76; 95% CI 0.62–0.92), with similar MACE benefits in patients without PAD (HR 0.86; 95% CI 0.76–0.98) No difference in amputation risk by PAD status (p_interaction_ of 0.31), but there was an overall increased risk of amputation with canagliflozin (HR 1.50; 95% CI 1.19–1.89). This was due to increase seen in CANVAS program
CANVAS & CANVAS-Renal & CREDENCE (canagliflozin)	Arnott et al. [34]	Post-hoc analysis of 3 double-blind, randomized trials, involving 10,142 patients with T2DM in CANVAS and 4401 patients with T2DM in CREDENCE, to determine if there was an explanation as to why the effects of canagliflozin on amputation risk vary between CANVAS and CREDENCE	Median follow-up was 2.4 years in CANVAS and 2.5 years in CREDENCE	There were 133 amputations in CREDENCE and 187 amputations in CANVAS, with prior amputation as strongest predictor of future amputations Effect of canagliflozin on amputation risk was significantly different between CANVAS and CREDENCE (p_heterogeneity_ of 0.02), but this was not explained by participant or trial differences. There was no evidence that foot disease management protocols in CREDENCE ameliorated amputation risk
DAPA-HF & DELIVER (dapagliflozin)	Butt et al. [15]	Post hoc analysis of 2 randomized, double-blind trials in 11,005 patients with symptomatic HF, 809 (7.4%) of whom with history of PAD, who received dapagliflozin (10 mg/day orally) vs. matching placebo	Median of 1.8 years	Dapagliflozin, compared to placebo, reduced risk of worsening HF or CV death to same extent in patients with (HR 0.71; 95% CI 0.54–0.94) and without (HR 0.80; 95% CI 0.73–0.88) PAD, with no interaction between PAD and effect of treatment (p_interaction_ of 0.39) Amputation rate did not differ between dapagliflozin and placebo in those with (HR 0.87; 95% CI 0.43–1.75) or without PAD (HR 0.87; 95% CI 0.46–1.64)
DECLARE-TIMI 58 (dapagliflozin)	Bonaca et al. [35]	Post hoc analysis of a double-blind, randomized trial involving 17,160 patients with T2DM and a history or a high risk of CV disease, including 1025 (6.0%) with a history of symptomatic lower extremity PAD, who received dapagliflozin (10 mg/day orally) vs. matching placebo	Median of 4.2 years	Patients in placebo arm with PAD vs. those without PAD had a higher adjusted risk of CV death/HHF (HR 1.60; 95% CI 1.21–2.12; *p* = 0.001), progression of kidney disease (HR 1.51; 95% CI 1.13–2.03; *p* < 0.01), and limb AEs (HR 8.37; 95% CI 6.45–10.87; *p* < 0.001) Overall, amputation risk was higher in those with vs. without PAD (5.6% vs. 1.1%; HR 4.47; 95% CI 2.86–7.00; *p* < 0.001). Predictors of amputation were PAD, longer T2DM duration, male sex, history of HF, higher baseline HbA1c, and non-use of statin and/or ezetimibe Benefit of dapagliflozin on HHF or CV death was consistent regardless of PAD status (PAD: HR 0.86; no PAD: HR 0.82; p_interaction_ of 0.79). Similarly, benefits for reductions in kidney complications with dapagliflozin vs. placebo were consistent (PAD: HR 0.78; no PAD: HR 0.76; p_interaction_ of 0.84) No differences between dapagliflozin vs. placebo in limb ischemic AEs (HR 1.07; 95% CI 0.90–1.26; *p* = 0.45) and amputation (HR 1.09; 95% CI 0.84–1.40; *p* = 0.53), with no significant interactions by presence of PAD or not (p_interaction_ of 0.30 and 0.093, respectively)
EMPA-REG OUTCOME (empagliflozin)	Verma et al. [16]	Post hoc analysis of a double-blind, randomized trial involving 7020 patients with T2DM and established CV disease, 1461 (20.8%) of whom had PAD at baseline, who received empagliflozin (10 or 25 mg/day orally) vs. placebo	Median of 3.1 years	In patients with PAD, empagliflozin vs. placebo reduced CV death by 43% (HR 0.57; 95% CI 0.37–0.88), all-cause death by 38% (HR 0.62; 95% CI 0.44–0.88), HHF by 44% (HR 0.56; 95% CI 0.35–0.92), and incident or worsening nephropathy by 46% (HR 0.54; 95% CI 0.41–0.71) In patients with PAD, rate of lower-limb amputations was 5.5% with empagliflozin and 6.3% with placebo (HR 0.84; 95% CI 0.54–1.32). In patients without PAD, rate of lower-limb amputations was 0.9% with empagliflozin and 0.7% with placebo (HR 1.30; 95% CI 0.69–2.4)
EMPA-REG OUTCOME (empagliflozin)	Inzucchi et al. [36]	Post hoc analysis of a double-blind, randomized trial in 7,020 patients with T2DM and CV disease, who received empagliflozin vs. placebo, aimed to assess lower-limb amputations in EMPA-REG OUTCOME	Median of 3.1 years	Lower-limb amputations were reported in 131 patients: 88/4,687 patients (1.9%) treated with empagliflozin and 43/2,333 (1.8%) treated with placebo. The incidence rate was 6.5 per 1000 patient-years in both groups. In the analysis of time to first event, the risk of lower-limb amputations was similar between empagliflozin and placebo (HR 1.00; 95% CI 0.70–1.44)
VERTIS CV (ertugliflozin)	Cannon et al. [37]	Double-blind, randomized trial in 8,246 patients with T2DM and CV disease, 1,541 (18.7%) of whom had PAD, who received ertugliflozin (5 or 15 mg/day orally) vs. placebo	Median of 3.0 years	MACE occurred in 653 of 5,493 patients (11.9%) in ertugliflozin group and in 327/2745 patients (11.9%) in placebo group (HR 0.97; 95% CI 0.85–1.11) Amputations were performed in 2.0% of ertugliflozin-treated patients and in 1.6% of patients receiving placebo. Vascular disorders occurred in 2.9% of ertugliflozin-treated patients and in 3.6% of patients receiving placebo
SOLOIST-WHF (sotagliflozin*)	Bhatt et al. [38]	Double-blind trial, randomizing 1,222 patients with T2DM and worsening HF to 200 or 400 mg of oral sotagliflozin or placebo once daily	Median of 9.0 months	Amputations were performed in 4/605 patients receiving sotagliflozin (0.7%) and 1/611 receiving placebo (0.2%)
SOTA-CKD3 (sotagliflozin*)	Cherney et al. [39]	Double-blind trial, randomizing 787 patients with T2DM and an eGFR of 30–59 ml/min/1.73 m^2^ to 200 or 400 mg of oral sotagliflozin or placebo once daily	52 weeks	MACE occurred in 18/527 patients (3.4%) receiving sotagliflozin and in 9/260 patients (3.5%) receiving placebo Amputations were performed in 3/527 patients (0.6%) receiving sotagliflozin and in 3/260 patients (1.2%) receiving placebo
SOTA-CKD4 (sotagliflozin*)	Cherney et al. [40]	Double-blind trial, randomizing 277 patients with T2DM and an eGFR of 15–30 ml/min/1.73 m^2^ to 200 or 400 mg of oral sotagliflozin or placebo once daily	52 weeks	MACE occurred in 7/184 patients (3.8%) receiving sotagliflozin and in 12/93 patients (12.9%) receiving placebo Amputations were performed in 3/184 patients (1.6%) receiving sotagliflozin and in 0/93 patients (0%) receiving placebo
*GLP-1RA*				
LEADER & SUSTAIN-6 (liraglutide and semaglutide)	Verma et al. [17]	Post hoc analysis of 2 randomized, double-blind trials in patients with T2DM at high CV risk or with CV disease. LEADER included 9,340 patients, 1,184 (12.7%) of whom had PAD, who received SC liraglutide (≤ 1.8 mg/day) vs. placebo. SUSTAIN-6 included 3,297 patients, 460 (14.0%) of whom had PAD, who received SC semaglutide (0.5 or 1.0 mg/week) vs. placebo	Median of 3.8 years in LEADER and 2.1 years in SUSTAIN-6	Patients with PAD were at a ~ 35% increased risk of MACE vs. those without PAD (LEADER: HR 1.36; 95% CI 1.17–1.58; *p* < 0.0001; SUSTAIN-6: HR 1.33; 95% CI 0.94–1.83; *p* = 0.09) Effects of both therapies on MACE were consistently beneficial in patients with PAD (liraglutide: HR 0.77; 95% CI 0.58–1.01; semaglutide: HR 0.61; 95% CI 0.33–1.13) and without PAD (liraglutide: HR 0.89; 95% CI 0.79–1.00; semaglutide: HR 0.77; 95% CI 0.58–1.01; p_interaction_ of 0.34 for liraglutide and 0.49 for semaglutide)
LEADER (liraglutide)	Dhatariya et al. [41]	Post hoc analysis of a double-blind, randomized trial in 9,340 patients with T2DM at high CV risk, aimed at assessing the impact of SC liraglutide (1.8 mg/day) vs. placebo on the incidence of DFUs and their sequelae	Median of 3.8 years	Similar rates of patients reported DFUs (176/4668 [3.8%] with liraglutide vs. 191/4,672 [4.1%] with placebo; HR 0.92; 95% CI 0.75–1.13; *p* = 0.41) Analysis of DFU-related sequelae demonstrated a significant reduction in amputations with liraglutide vs. placebo (HR 0.65; 95% CI 0.45–0.95; *p* = 0.028). However, there was no difference between treatments in DFU requiring peripheral revascularization (HR 0.87; 95% CI 0.48–1.58; *p* = 0.64)
EXSCEL (exenatide)	Badjatiya et al. [18]	Post hoc analysis of a double-blind, randomized trial involving 14,752 patients with T2DM, 2,800 (19.0%) of whom had documented PAD, who received SC exenatide (2 mg/week) vs. placebo	Median of 3.2 years	Patients with PAD were less likely to be on a statin (65.8% vs. 75.3%), a β-blocker (45.4% vs. 58.1%), an angiotensin-converting enzyme inhibitor (45.2% vs. 49.5%), or aspirin (57.3% vs. 65.0%) vs. patients without PAD Compared to patients without PAD, those with PAD had higher rates of MACE (13.6% vs. 11.4%; HR 1.13; 95% CI 1.00–1.27; *p* = 0.047), all-cause mortality (10.0% vs. 6.8%; HR 1.38; 95% CI 1.20–1.60; *p* < 0.001), and amputations (5.0% vs. 0.9%; HR 5.48; 95% CI 4.16–7.22; *p* < 0.001) Exenatide and placebo resulted in similar rates of amputations in those with PAD (5.0% with exenatide vs. 4.9% with placebo; HR 0.99; 95% CI 0.71–1.38) and in those without PAD (0.9% in both groups; HR 0.96; 95% CI 0.66–1.41; p_interaction_ of 0.92). Patients treated with exenatide or placebo also had similar rates of MACE, regardless of PAD status (p_interaction_ of 0.42)

AE, adverse event; CANVAS, CANagliflozin cardioVascular Assessment Study; CI, confidence interval; CKD, chronic kidney disease; CREDENCE, Canagliflozin and Renal Events in Diabetes with Established Nephropathy Clinical Evaluation; CV, cardiovascular; DAPA-HF, Dapagliflozin and Prevention of Adverse Outcomes in Heart Failure; DECLARE-TIMI 58, Dapagliflozin Effect on Cardiovascular Events-Thrombolysis in Myocardial Infarction 58; DELIVER, Dapagliflozin Evaluation to Improve the Lives of Patients With Preserved Ejection Fraction Heart Failure; DFU, diabetic foot ulcer; eGFR, estimated glomerular filtration rate; EMPA-REG OUTCOME, Empagliflozin Cardiovascular Outcome Event Trial in Type 2 Diabetes Mellitus Patients; ESKD, end-stage kidney disease; EXSCEL, Exenatide Study of Cardiovascular Event Lowering; HbA1c, glycated hemoglobin; HF, heart failure; HHF, hospitalization for heart failure; HR, hazard ratio; LEADER, Liraglutide Effect and Action in Diabetes: Evaluation of Cardiovascular Outcome Results; MACE, major adverse cardiovascular events; SC, subcutaneous; SOLOIST-WHF, Effect of Sotagliflozin on Cardiovascular Events in Patients with Type 2 Diabetes Post Worsening Heart Failure; SUSTAIN-6, Trial to Evaluate Cardiovascular and Other Long-term Outcomes with Semaglutide in Subjects with Type 2 Diabetes; T2DM, type 2 diabetes mellitus; VERTIS CV, Evaluation of Ertugliflozin Efficacy and Safety Cardiovascular Outcomes; vs., versus

*Sotagliflozin is a dual inhibitor of SGLT1 and SGLT2

### How does T2DM affect the pathophysiology of PAD?

The pathophysiology of PAD in patients with T2DM is similar to that in the non-diabetic population, except that the presence of concomitant diabetes mellitus can potentiate and accelerate the development and progression of PAD [9, 42]. The underlying T2DM metabolic abnormalities, namely chronic hyperglycemia, insulin resistance, and dyslipidemia, promote vascular inflammation, endothelial cell dysfunction, vasoconstriction, platelet activation, and thrombosis, all of which contribute to the progression of atherosclerotic lesions as well as microvascular damage in patients with T2DM [9, 42–45]. Endothelial dysfunction in T2DM can also be attributed to an overproduction of vasoconstrictors (e.g., endothelin-1) and prostanoids (e.g., thromboxane A2), contributing to abnormal vascular smooth muscle cell growth and migration [9, 46].

T2DM is considered as a proinflammatory state, associated with elevated levels of C-reactive protein and proinflammatory cytokines [9, 20, 47, 48]. This is further compounded by hyperglycemia-induced activation of inflammatory pathways, which leads to the development of atherosclerosis [45, 46, 49]. T2DM is additionally associated with the enhanced production of advanced glycation end products that interact with their receptors to upregulate inflammatory transcription factors, leading to medial calcification and an increased leukocyte activity [43, 45, 49, 50]. Likewise, T2DM potentiates platelet aggregation, accelerates platelet turnover, and heightens blood coagulability by increasing the expression of tissue factor and decreasing antithrombin levels, contributing to a thrombotic environment [9, 51].

**Fig. 1:**
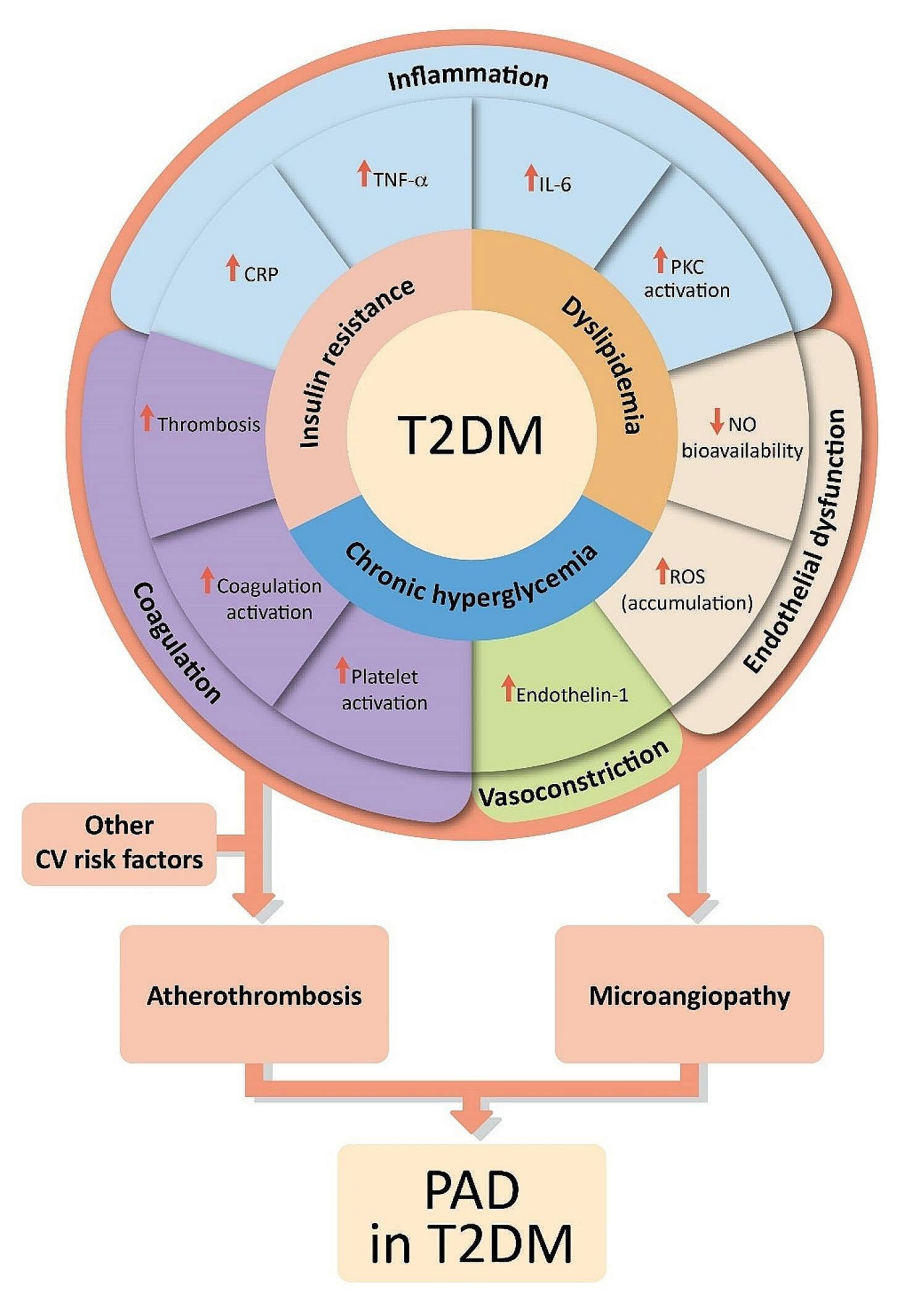
Pathophysiology of lower extremity peripheral artery disease (PAD) in patients with type 2 diabetes mellitus (T2DM). Other cardiovascular (CV) risk factors may include advanced age, smoking, hypertension, longer duration of diabetes, neuropathy, retinopathy, and prior history of CV disease. *Abbreviations* CRP, C-reactive protein; IL, interleukin; NO, nitric oxide; PKC, protein kinase C; ROS, reactive oxygen species; TNF-α, tumor necrosis factor-alpha

Overall, the interplay of all these aforementioned factors in individuals with T2DM accelerates the development and progression of atherosclerosis, which, coupled with diabetic microvascular complications, worsens the prognosis of PAD in the lower extremities (Fig. 1).

### Features of PAD in patients with T2DM

Table 3 compares the typical features of lower extremity PAD in individuals with T2DM to those without T2DM. Compared to non-diabetic PAD, T2DM is associated with more distal lesions, and a more diffuse and multisegmental pattern of PAD [2, 52, 53].

**Table 3:** Characteristics of peripheral artery disease (PAD) in patients with type 2 diabetes mellitus (T2DM) compared with patients without T2DM

Patients with PAD without T2DM	Patients with PAD and T2DM
*Anatomical aspects* [2, 50, 53, 54]	
Anatomical localization mainly proximal (aorta, iliac, femoral, and popliteal arteries)	Anatomical localization mainly distal (popliteal, tibial, and fibular arteries)
Focal pattern of PAD	Diffuse, multisegmental, and bilateral pattern of PAD
Less extensive arterial wall calcification, which is often more localized and intimal	Extensive arterial wall calcification often observed, which is more circumferential and medial
Collateral arterial bed rather well-developed	Impaired collateral arterial bed often observed
*Clinical features* [2, 9, 20, 53, 55]	
Typical PAD presentation such as intermittent claudication	More frequent asymptomatic presentation or atypical symptoms such as leg fatigue or slow walking velocity
Individuals are generally more aware of foot wounds	Higher risk of non-healing foot wounds, consequently leading to an increased risk of infections
Progression may be slow and often correlates more directly with lifestyle factors (e.g., smoking, hyperlipidemia)	Faster progression with a higher risk of gangrene, chronic limb-threatening ischemia, and amputations
Ankle-brachial index (ABI) and other non-invasive tests are more reliable for diagnosing PAD	Due to medial arterial calcification, ABI may be falsely elevated (underdiagnosis)
Treatment response can be more predictable	Might have a less favorable response to certain treatments (e.g., angioplasty), and may require more aggressive medical management

In patients with T2DM, PAD is commonly asymptomatic due to the presence of diabetic neuropathy [20]. This concomitant peripheral neuropathy may predispose patients with T2DM and PAD to present with advanced disease compared to patients without diabetes [2, 55]. Hence, those with T2DM and PAD are more likely to develop chronic limb-threatening ischemia (CLTI) [5, 11, 56]. The coexistence of diabetes mellitus with peripheral neuropathy and PAD may also make the presentation of foot infection more subtle [11]. Besides neuropathy, other diabetic microvascular complications such as diabetic retinopathy are also associated with more severe PAD and CLTI [55, 57].

### Pitfalls in the diagnosis of PAD in T2DM

Various clinical practice guidelines recommend the annual examination of all patients with T2DM for the presence of PAD, even in the absence of foot ulceration. This examination should include a medical history, assessing exertional leg symptoms (intermittent claudication or other walking impairment, ischemic rest pain, and non-healing wounds), palpating peripheral pulses, and examining the skin’s appearance (color, temperature, and pilosity) [11, 13, 23, 25, 26, 58, 59]. Indeed, performing a thorough skin examination is important in patients with T2DM, as there are diabetic cutaneous manifestations associated with PAD, most commonly diabetic dermopathy [60]. In addition, features such as dry, cool, or fissured skin, absence of hair growth, and dystrophic toenails are frequently observed in patients with PAD [61]. Neuropathy, which is a major risk factor for tissue loss, should also be assessed using 10-g monofilaments and, if available, a tuning fork to assess vibration sense [26, 61]. Overall, such a thorough clinical evaluation is essential to detect masked PAD in patients with T2DM [26].

In patients with clinical suspicion for PAD (e.g., in case of absent or diminished foot pulses), the diagnosis of PAD is established with the measurement of the resting ABI. Patients with ABI ≤ 0.90 are diagnosed with PAD [4, 11, 12, 31]. However, although ABI is currently the first choice for evaluating PAD, peripheral diabetic arteries frequently have medial and intimal calcifications, resulting in higher segmental and ankle pressures and consequently an elevated ABI (> 1.40) [52]. A retrospective study including 1162 patients with symptomatic PAD from a United States vascular laboratory showed that resting ABI had a reduced accuracy of 66% in patients with diabetes versus 81% in patients without diabetes (*p* < 0.001) [62]. Hence, in patients with T2DM, it is recommended to also measure the TBI and toe pressure, because medial calcification rarely affects digital arteries. A TBI ≤ 0.70 is diagnostic of PAD [4, 11]. The toe pressure is normally 10 mmHg lower than the brachial pressure, a toe pressure < 40 mmHg predicts impaired wound healing for ischemic ulcers, and a toe pressure < 30 mmHg can be used as a hemodynamic diagnostic criterion for CLTI [52, 61]. Transcutaneous oxygen pressure (TcPO_2_) at rest or during exercise is another measure of skin perfusion that is not affected by calcification of the medial arteries, and can thus be also useful in patients with T2DM. A resting TcPO_2_ value < 30 mmHg can be used as a hemodynamic diagnostic criterion for CLTI [31].

**Fig. 2:**
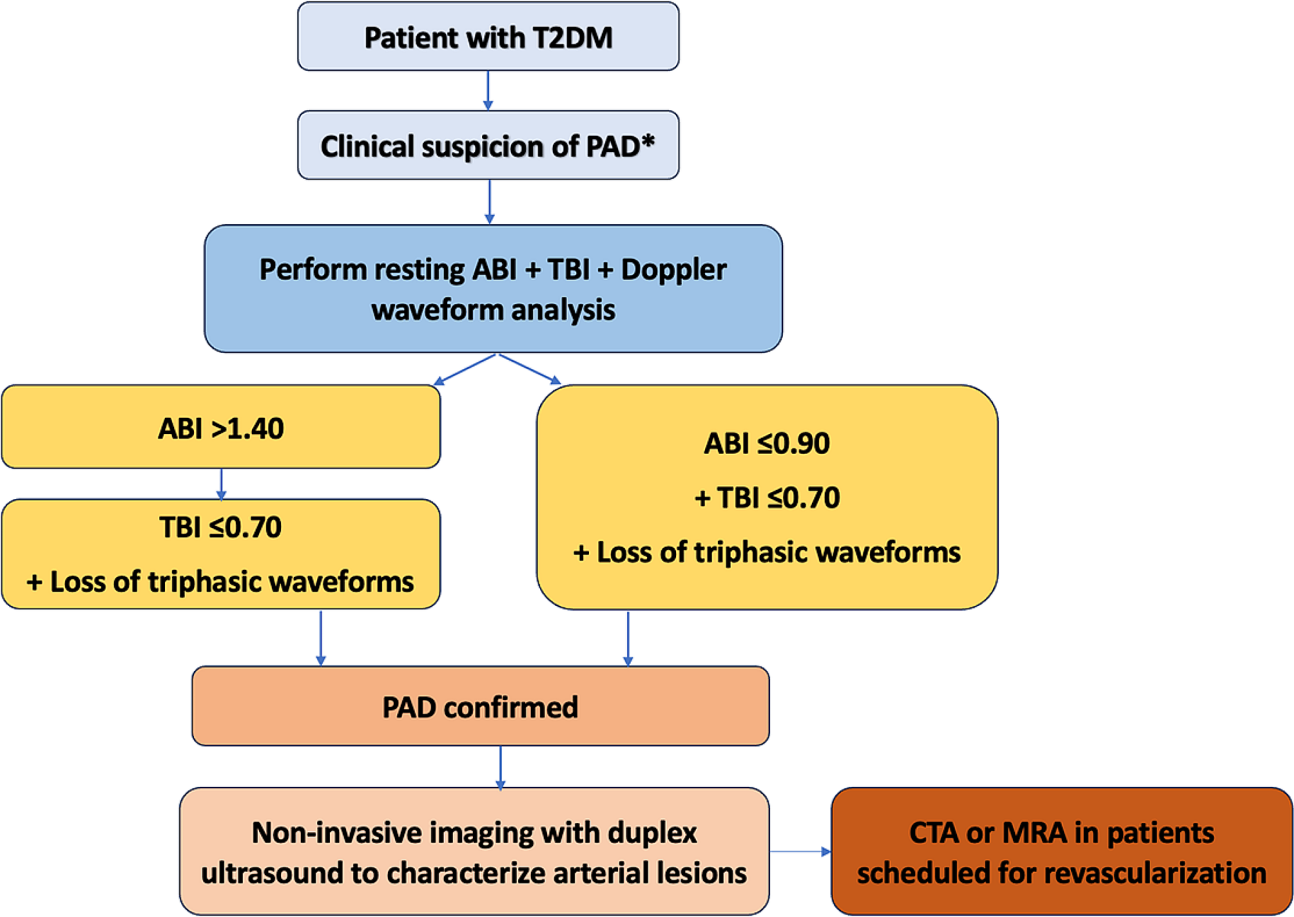
Diagnostic approach for lower extremity peripheral artery disease (PAD) in patients with type 2 diabetes mellitus (T2DM). *Abbreviations* ABI, ankle-brachial index; CTA, computed tomography angiography; MRA, magnetic resonance angiography; TBI, toe-brachial index. *Recommended annual clinical evaluation (medical history, feet inspection, assessing PAD symptoms, monofilament test)

In addition to measuring the ABI and the TBI, Doppler waveform analysis of the ankle arteries is recommended in patients with T2DM and suspected PAD to detect occlusive disease despite calcified arteries [4, 26, 31]. In a retrospective, community-based study from Australia performed in 396 patients with suspected PAD, which used color duplex ultrasound as the reference standard, the sensitivity of continuous-wave Doppler waveform analysis was unaffected by the presence of diabetes (83% in patients with diabetes and 81% in those without diabetes) [63]. Similarly, the specificity of continuous-wave Doppler was unaffected by diabetes (88% in patients with diabetes and 90% in those without diabetes) [63]. Doppler waveform analysis has also been found to be useful in evaluating PAD severity and for the detection of CLTI [64]. In patients with T2DM with confirmed PAD by an ABI ≤ 0.90, a TBI ≤ 0.70, and/or monophasic/biphasic Doppler waveform morphology, additional non-invasive imaging with duplex ultrasound, magnetic resonance angiography, or computed tomographic angiography can be performed to characterize the arterial lesions present and to develop an individualized treatment plan [4, 11, 31]. Figure 2 summarizes our overall diagnostic algorithm of PAD in patients with T2DM.

To aid in the early detection of PAD in individuals with T2DM, the American Diabetes Association recommends screening for asymptomatic PAD using the ABI in patients with T2DM at high risk for PAD, including any of the following: age ≥ 50 years, diabetes duration ≥ 10 years, comorbid microvascular disease, clinical evidence of foot complications, or any end-organ damage from diabetes [21]. However, the usefulness of screening PAD using the ABI and the TBI among patients with T2DM without any symptoms or wound problems remains a topic of debate. There are no randomized trials comparing PAD screening versus no screening in patients with T2DM. Moreover, the United States Preventive Services Task Force suggested that in patients with T2DM who are already at high risk for cardiovascular disease (CVD), screening for PAD with an ABI is unlikely to alter effective management decisions and improve clinical outcomes [65]. Nevertheless, screening for PAD using the ABI is justifiable in patients with T2DM, given that PAD is a public health issue that is often underrecognized, and not performing this non-invasive and readily available diagnostic test is potentially harmful in individuals at high risk for PAD [66].

### Does T2DM make a difference in the therapeutic approach of PAD?

#### What do treatment guidelines recommend?

The therapeutic approach of PAD in individuals with T2DM is consistent across different clinical practice guidelines, as summarized in Table 1. Given that the combination of T2DM and PAD is associated with a very high cardiovascular risk [67], general cardiovascular prevention is of the utmost importance and encompasses non-pharmacological measures such as smoking cessation, a healthy diet, and structured exercise [12, 13]. In addition, pharmacological therapy includes antihypertensive drugs, lipid-lowering agents, glucose-lowering agents, and antithrombotic agents [23, 58].

It is recommended to target systolic blood pressure between 120 and 130 mmHg and diastolic blood pressure below 80 mmHg in patients with T2DM, while avoiding orthostatic hypotension in older (> 65 years) and frail patients [26]. Aggressive management of dyslipidemia in patients with T2DM and PAD is also necessary, with a ≥ 50% reduction from baseline for low-density lipoprotein (LDL) cholesterol and a recommended target value < 1.4 mmol/L (< 55 mg/dL) [26]. Regardless of baseline LDL cholesterol levels, clinical practice guidelines recommend statin therapy in all patients with PAD for the prevention of major adverse cardiovascular events (MACE) (cardiovascular death, non-fatal myocardial infarction, and non-fatal stroke) and major adverse limb events (MALE) (limb ischemia, amputation, or PAD-related revascularization) [11–13, 23, 24, 29, 30]. On top of general prevention, statins are also indicated in patients with PAD to improve walking distance [11, 12, 68]. In patients with PAD who do not achieve their target LDL cholesterol on statin therapy alone, additional lipid-lowering therapy with ezetimibe (a cholesterol absorption inhibitor) and a proprotein convertase subtilisin/kexin type 9 (PCSK9) inhibitor is recommended [21, 26]. PCSK9 inhibition has been found to significantly reduce the risk of MACE in patients with PAD [69, 70].

As a secondary prophylaxis in patients with T2DM and symptomatic PAD, all clinical practice guidelines advocate single antiplatelet therapy, either aspirin (75–100 mg per day) or clopidogrel (75 mg per day), to reduce the risk of MACE [11–13, 24, 29, 30]. Of note, long-term dual antiplatelet therapy (aspirin plus clopidogrel) is not recommended in patients with T2DM and symptomatic PAD, as it may increase the risk of bleeding without providing substantial additional cardiovascular benefits [11, 12]. However, a combination of low-dose rivaroxaban (2.5 mg twice daily) and aspirin (100 mg once daily) should be considered in patients with symptomatic PAD at a low risk of bleeding [26, 27]. Compared with aspirin alone, the addition of rivaroxaban to aspirin reduced the risk of MACE and MALE in patients with symptomatic PAD [71–73].

When intermittent claudication impairs everyday life activities, or if a patient with T2DM and symptomatic PAD develops CLTI, revascularization is recommended to restore direct blood flow to at least one of the foot arteries [11, 12, 20, 23, 24, 29, 30]. Importantly, any revascularization procedure should be part of a comprehensive care plan that addresses other issues encountered in patients with T2DM and PAD including: prompt treatment of any concurrent foot infection, regular wound debridement, biomechanical offloading (if inappropriate plantar pressures are detected), control of blood glucose, assessment and improvement of nutritional status, treatment of edema and other comorbidities, as well as exercise rehabilitation [11, 13, 74].

### Role of glucose-lowering agents in T2DM and PAD

The risk of both microvascular and macrovascular complications of T2DM is strongly associated with hyperglycemia [75]. In the EUCLID (Examining Use of tiCagreLor In peripheral artery Disease) trial, every 1% increase in glycated hemoglobin (HbA1c) was associated with a 14% increased risk for MACE in patients with symptomatic PAD and T2DM [76]. Hence, the achievement of a HbA1c level < 7.0% (< 53 mmol/mol) is recommended in patients with T2DM and PAD to reduce microvascular complications, and should be considered for reducing macrovascular complications in the long term [11, 12, 20, 24, 26]. Target HbA1c levels should nevertheless be individualized in accordance with age, T2DM duration, and patient comorbidities, while avoiding hypoglycemic episodes [26].

The choice of glucose-lowering agents in patients with PAD should also be individualized to the key product characteristics, the patient’s wishes, preferences, and financial support/drug coverage [24]. However, it is recommended to include GLP-1RA or SGLT2i in the medical management of patients with T2DM and PAD, since they have demonstrated cardiovascular benefits [22, 26, 29, 30]. GLP-1RA show in particular great promise for treating PAD in patients with T2DM, since they may have systemic microcirculatory benefits in the peripheral vascular district, including reduced inflammation and oxidative stress, improved endothelial function, vasodilatation, and anti-atherosclerotic effects [77–82]. In a recent open-label RCT of 55 patients with T2DM and PAD, the administration of liraglutide improved peripheral perfusion, suggesting that it may prevent the clinical progression of PAD [83]. The main mechanisms supporting the cardiorenal protective effects of SGLT2i include the correction of cardiorenal risk factors, metabolic adjustments ameliorating myocardial substrate utilization, and optimization of ventricular loading conditions through effects on diuresis, natriuresis, and vascular function [84, 85]. Both GLP-1RA and SGLT2i are well-tolerated, with gastrointestinal symptoms and polyuria being the most common side effects of GLP-1RA and SGLT2i, respectively [86]. They are also associated with weight loss, which is mainly due to loss of fat mass. However, the concomitant loss of lean mass warrants attention and requires prevention strategies to preserve skeletal muscle and improve physical function [87].

Of note, before the breakthrough of GLP-1RA and SGLT2i, a few studies showed that metformin reduced the risk of MALE and MACE in patients with T2DM [88, 89], including in those with PAD [90]. However, the same risk reduction of MACE was found in patients with T2DM treated with dulaglutide (a GLP-1RA) and metformin compared to those treated with dulaglutide alone, questioning the need for metformin [91, 92].

CVOT analyses provide insights into the cardiovascular benefits and safety profile of GLP-1RA and SGLT2i in PAD (Table 2). Importantly, SGLT2i have been shown to be beneficial in patients with chronic kidney disease (CKD) and/or heart failure—two frequent comorbidities in patients with PAD—regardless of the presence of diabetes [14, 15]. However, in a recent meta-analysis of 20 RCTs evaluating the effectiveness of SGLT2i in reducing the risk of PAD in 59,952 patients with T2DM, the use of SGLT2i did not significantly change the incidence of PAD compared to placebo or oral glucose-lowering agents (relative risk [RR], 0.98; 95% confidence interval [CI] 0.78–1.24) [93]. Subgroup analysis further revealed that the risk of incident PAD did not differ between the four evaluated SGLT2i: canagliflozin (RR, 1.18; 95% CI 0.70–1.99), dapagliflozin (RR, 0.86; 95% CI 0.58–1.27), empagliflozin (RR, 1.16; 95% CI 0.75–1.79), and ertugliflozin (RR, 0.83; 95% CI 0.49–1.40) [93]. SGLT2i were also not associated with an increased risk of restenosis in a real-world study from Japan among 157 patients with T2DM undergoing femoropopliteal endovascular therapy with drug coated balloon for symptomatic PAD [94].

Regarding the safety of SGLT2i, in a real-world study using three nationwide United States databases, including 96,128 adults with CKD and T2DM who newly filled prescriptions for SGLT2i versus GLP-1RA, SGLT2i compared with GLP-1RA were associated with a higher risk of lower-limb amputations (hazard ratio [HR], 1.65; 95% CI 1.22–2.23) and of non-vertebral fractures (HR 1.30; 95% CI 1.03–1.65) [95]. Moreover, in the CANVAS (CANagliflozin cardioVascular Assessment Study) program including 10,142 patients with T2DM at high cardiovascular risk, canagliflozin was associated with a 1.97-fold increased risk (95% CI 1.41–2.75) of lower-limb amputations [32]. Identified independent predictors of amputation were prior amputations, male sex, non-Asian ethnicity, history of peripheral vascular disease, history of neuropathy, albuminuria, and increased HbA1c at baseline [32]. However, in the CREDENCE (Canagliflozin and Renal Events in Diabetes with Established Nephropathy Clinical Evaluation) trial including 4,401 patients with T2DM and CKD, similar amputation rates were found in the canagliflozin and placebo groups (HR 1.11; 95% CI 0.79–1.56) [96]. Moreover, no increased amputation risk was observed in CVOTs using other SGLT2i [15, 16, 35–38, 97].

**Fig. 3:**
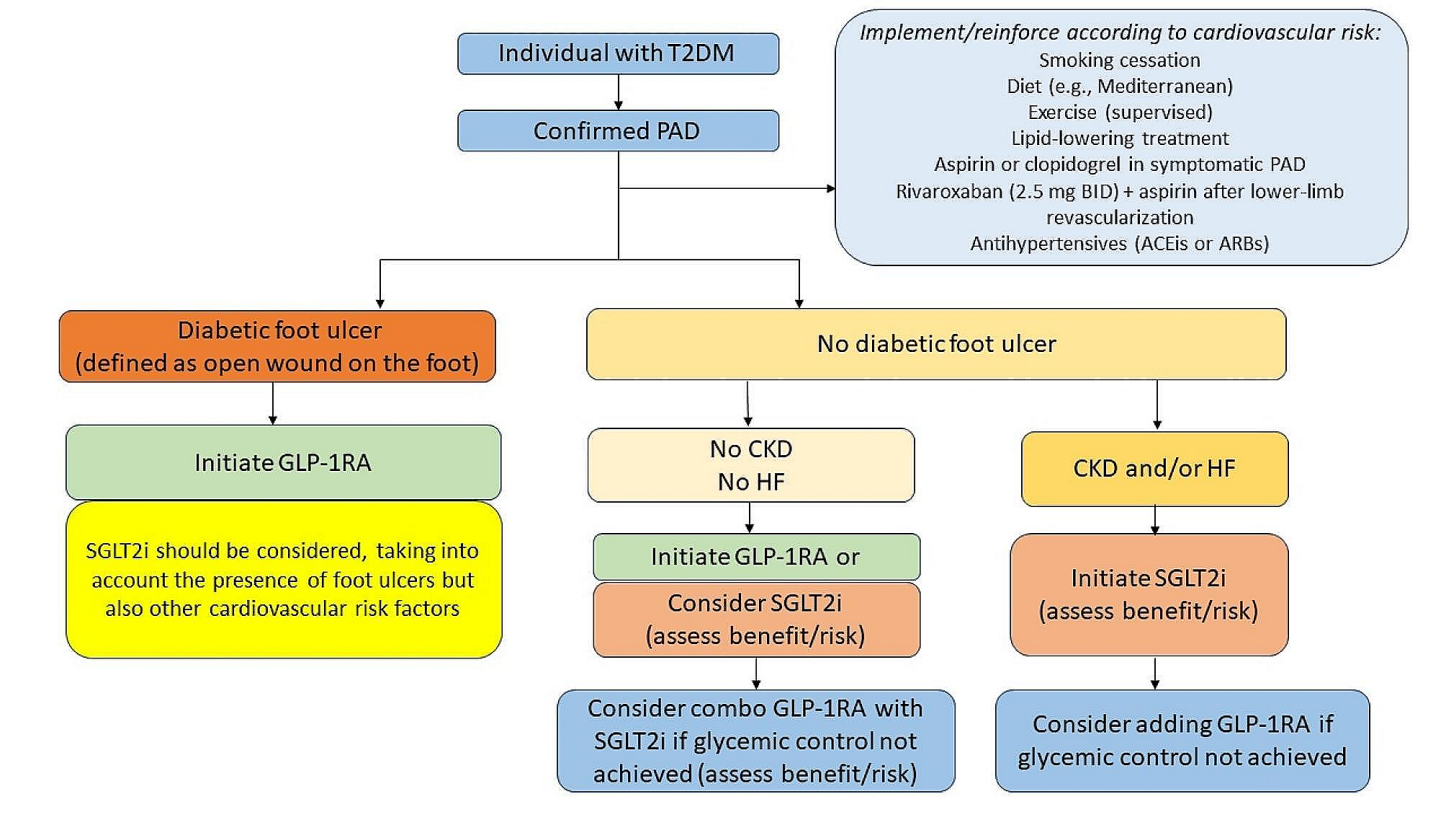
Glucose-lowering management approach for lower extremity peripheral artery disease (PAD) in patients with type 2 diabetes mellitus (T2DM). Abbreviations: ACEi, angiotensin-converting enzyme inhibitor; ARB, angiotensin-receptor blocker; BID, twice daily; CKD, chronic kidney disease; GLP-1RA, glucagon-like peptide-1 receptor agonist; HF, heart failure; SGLT2i, sodium-glucose cotransporter-2 inhibitor

Overall, it is advisable to conduct a thorough screening for risk factors for amputations when initiating SGLT2i. These risk factors include a history of amputations, neuropathy, high HbA1c at baseline, and diabetic foot ulcers (DFUs) [32]. It is also advised to be cautious with the use of SGLT2i in patients with an active DFU and to carefully weigh the individual benefit-risk balance (Fig. 3). Recent evidence also highlights an increased risk of amputation in patients with PAD or at high risk for PAD who are under diuretics [98]. Thus, the addition of SGLT2i on top of diuretics should be discussed case by case.

Comparisons between GLP-1RA and SGLT2i are scarce, with no available data from RCTs. The impact of GLP-1RA on the progression of PAD in patients with T2DM was evaluated in real-world studies [99, 100]. Compared to SGLT2i, the use of GLP-1RA was associated with a significantly lower risk of MALE, which was driven by a lower incidence of CLTI (HR 0.83; 95% CI 0.68–1.02) [100].

In terms of CVOT findings, a post-hoc analysis of LEADER (Liraglutide Effect and Action in Diabetes: Evaluation of Cardiovascular Outcome Results), performed in 9340 patients with T2DM at high cardiovascular risk, found that treatment with liraglutide did not increase the risk of DFUs (defined as an open wound on the foot) and was associated with a significantly lower risk of DFU-related amputations compared to placebo (HR 0.65; 95% CI 0.45–0.95) [41]. Semaglutide was also associated with a lower need for coronary and peripheral revascularization compared to placebo (HR 0.65; 95% CI 0.50–0.86) in SUSTAIN-6 (Trial to Evaluate Cardiovascular and Other Long-term Outcomes with Semaglutide in Subjects with Type 2 Diabetes) conducted in 3297 patients with T2DM at high cardiovascular risk [101]. In a more recent post-hoc analysis of both LEADER and SUSTAIN-6, liraglutide and semaglutide reduced MACE, with consistent cardiovascular efficacy regardless of PAD status [17]. EXSCEL (Exenatide Study of Cardiovascular Event Lowering) is another CVOT evaluating exenatide in 14,752 patients with T2DM with or without CVD including PAD [102]. Treatment with exenatide or placebo resulted in similar rates of non-traumatic amputations in those with PAD (HR 0.99; 95% CI 0.71–1.38) and in those without PAD (HR 0.96; 95% CI 0.66–1.41). Exenatide was also associated with a significantly lower all-cause mortality in patients with T2DM and PAD (HR 0.77; 95% CI 0.61–0.98) [18].

STRIDE is an ongoing trial (NCT04560998) that is investigating the effect of subcutaneous once-weekly semaglutide on walking ability compared to placebo in patients with T2DM and symptomatic PAD with intermittent claudication. STRIDE is expected to provide valuable insights into the functional outcomes of GLP-1RA for individuals with T2DM and PAD. Similarly, additional data is anticipated from the long-term placebo-controlled SOUL trial (NCT03914326) investigating the effects of oral semaglutide on MACE and MALE in patients with T2DM and CKD or CVD, including individuals with symptomatic PAD.

Figure 3 summarizes our directions in the pharmacological treatment algorithm of PAD in patients with T2DM, including the incorporation of GLP-1RA and SGLT2i. Of note, based on individual metabolic control and cardiovascular and renal risk factors, the association of a GLP-1RA with a SGLT2i could be considered [22, 103]. Although SGLT2i in patients with CKD or heart failure could be beneficial, it is essential that when initiating SGLT2i alone or in combination with GLP-1RA, a thorough individual evaluation of the benefit-risk profile is conducted to mitigate any potential risk of amputations.

### Perspectives for optimizing PAD management in patients with T2DM

Despite the availability of various clinical practice guidelines for the therapeutic management of patients with T2DM and PAD and their overall consistency, there can be gaps in their implementation in real-life clinical practice [104]. Suboptimal rates of evidence-based therapies have also been noted among patients with T2DM and PAD. In a real-world analysis of a large claims database from the United States, performed in 543,938 patients with T2DM and atherosclerotic CVD, including 294,092 (54.1%) patients with PAD, the use of GLP-1RA and SGLT2i was found to be low (< 9%) [105]. Similarly, CAPTURE, a non-interventional, cross-sectional, multinational study conducted in 9823 adults with T2DM (36.5% with CVD including PAD), revealed that GLP‐1RA and/or SGLT2i were used by 21.9% of participants, with comparable rates among patients with and without CVD (21.5% and 22.2%, respectively) [106]. Furthermore, in a meta-analysis of 86 studies investigating the rates of prescription of vasculoprotective therapies in patients with PAD, the pooled literature estimates for the utilization of antiplatelets, statins, and angiotensin-converting enzyme inhibitors/angiotensin receptor blockers were 75%, 56%, and 53%, respectively, indicating important treatment gaps [107].

Efforts to bridge these treatment gaps can include continuing medical education for healthcare providers, as well as patient education. A barrier to the initiation of novel glucose-lowering therapies, particularly GLP-1RA, is their administration via injections. There is hence a necessity to encourage pharmacists and nurse practitioners to offer patient education on these injectable treatments. Patients should also be educated on the importance of inspecting their feet daily, proper footwear, proper nail hygiene, and the importance of seeking medical attention for any foot problems like cuts, sores, or changes in the color or temperature of the feet [11].

The optimal management of PAD in patients with T2DM requires a dedicated multidisciplinary collaboration, involving endocrinologists/diabetologists, vascular surgeons, cardiologists, podiatrists, primary care specialists, and other healthcare professionals [13, 25]. Nather et al. [108] from Singapore evaluated the effectiveness of a hospital multidisciplinary team in improving the management of diabetic foot problems. They found that the introduction of a multidisciplinary team reduced the average length of hospital stay from 20.4 to 12.2 days and the major amputation rate from 31.2 to 11.0%. In a similar study from China, the introduction of a multidisciplinary team, coordinated by an endocrinologist and a podiatrist for managing diabetic foot problems, was associated with a reduction in the frequency of major amputations from 9.5 to < 5% [109]. Overall, both studies highlight the effectiveness of a multidisciplinary approach in improving patient care, reducing complications, and potentially saving healthcare costs [108, 109]. It is also important that all patients with T2DM, even those without a DFU, have their peripheral arteries examined at least annually through a medical history and pedal pulse palpation [110].

### Strengths and limitations

This review article is strengthened by the inclusion of multiple data sources, incorporating both clinical practice guidelines and RCTs/CVOTs, to provide a comprehensive overview of the current evidence on PAD management in patients with T2DM. In addition, the conducted systematic searches were thorough and industry-independent, ensuring a broad and unbiased inclusion of relevant literature. Nevertheless, as with any literature review, there is a risk of publication bias. Moreover, the quality of the included studies can vary, which can affect the overall strength of the evidence presented.

## Conclusions

PAD, characterized by atherosclerosis in the arteries of the lower extremities, is highly prevalent in patients with T2DM. The management of PAD in patients with T2DM requires a multidisciplinary and individualized approach that addresses both the overarching metabolic disturbances inherent to diabetes and the specific vascular complications of PAD. While there are several societal guidelines for the diagnosis and treatment of PAD in patients with T2DM, it is important to acknowledge that these guidelines are primarily based on data from the general population. To better tailor recommendations and improve care for this particular population, there is a pressing need for robust, T2DM-specific PAD clinical trials, primarily focusing on novel glucose-lowering agents known for their cardiovascular benefits, including GLP-1RA and SGLT2i. The initiation of such focused research efforts is essential to inform and refine clinical practices, optimizing patient outcomes in this complex interplay of systemic metabolic dysfunction and localized vascular impairment.

### Electronic supplementary material

Below is the link to the electronic supplementary material.

Supplementary Material 1.

## Data Availability

No datasets were generated or analysed during the current study.

## Abbreviations

ABI: Ankle-brachial index
ACC: American College of Cardiology
ACEi: Angiotensin-converting enzyme inhibitor
ADA: American Diabetes Association
ADFDG: Australian Diabetes-related Foot Disease Guidelines
AE: Adverse event
AHA: American Heart Association
ARB: Angiotensin-receptor blocker
CANVAS: CANagliflozin cardioVascular Assessment Study
CCS: Canadian Cardiovascular Society
CDS: Chinese Diabetes Society
CI: Confidence interval
CKD: Chronic kidney disease
CLTI: Chronic limb-threatening ischemia
CREDENCE: Canagliflozin and Renal Events in Diabetes with Established Nephropathy Clinical Evaluation
CRP: C-reactive protein
CVD: Cardiovascular disease
CVOT: Cardiovascular outcome trial
DAPA-HF: Dapagliflozin and Prevention of Adverse Outcomes in Heart Failure
DECLARE-TIMI 58: Dapagliflozin Effect on Cardiovascular Events-Thrombolysis in Myocardial Infarction 58
DELIVER: Dapagliflozin Evaluation to Improve the Lives of Patients With Preserved Ejection Fraction Heart Failure
DFU: Diabetic foot ulcer
eGFR: Estimated glomerular filtration rate
EMPA-REG OUTCOME: Empagliflozin Cardiovascular Outcome Event Trial in Type 2 Diabetes Mellitus Patients
ESC: European Society of Cardiology
ESKD: End-stage kidney disease
ESVS: European Society for Vascular Surgery
EUCLID: Examining Use of tiCagreLor In peripheral artery Disease
EXSCEL: Exenatide Study of Cardiovascular Event Lowering
GDS: German Diabetes Society
GLP-1RA: Glucagon-like peptide-1 receptor agonist
HbA1c: Glycated hemoglobin
HF: Heart failure
HHF: Hospitalization for heart failure
HR: Hazard ratio
IL: Interleukin
LDL: Low-density lipoprotein
LEAD: Lower extremity arterial disease
LEADER: Liraglutide Effect and Action in Diabetes: Evaluation of Cardiovascular Outcome Results
MACE: Major adverse cardiovascular events
MALE: Major adverse limb events
NO: Nitric oxide
PAD: Peripheral artery disease
PCSK9: Proprotein convertase subtilisin/kexin type 9
PKC: Protein kinase C
RCT: Randomized controlled trial
ROS: Reactive oxygen species
RR: Relative risk
SC: Subcutaneous
SCVE: French Society for Vascular and Endovascular Surgery
SFMV: French Society of Vascular Medicine
SGLT2i: Sodium-glucose cotransporter-2 inhibitor
SOLOIST-WHF: Effect of Sotagliflozin on Cardiovascular Events in Patients with Type 2 Diabetes Post Worsening Heart Failure
SUSTAIN-6: Trial to Evaluate Cardiovascular and Other Long-term Outcomes with Semaglutide in Subjects with Type 2 Diabetes
T2DM: Type 2 diabetes mellitus
TBI: Toe-brachial index
TcPO_2_: Transcutaneous oxygen pressure
TNF-α: Tumor necrosis factor-alpha
VERTIS CV: Evaluation of Ertugliflozin Efficacy and Safety Cardiovascular Outcomes
vs.: Versus
