# Supplementary material for: Challenges and opportunities in the management of type 2 diabetes in patients with lower extremity peripheral artery disease: a tailored diagnosis and treatment review
Source: Cardiovasc Diabetol. 2024 Jun 26;23:220. doi: 10.1186/s12933-024-02325-9 (PMC11210102; doi:10.1186/s12933-024-02325-9)
Supplement: Supplementary file 1 — Supplementary Material 1. [file 12933_2024_2325_MOESM1_ESM.docx]

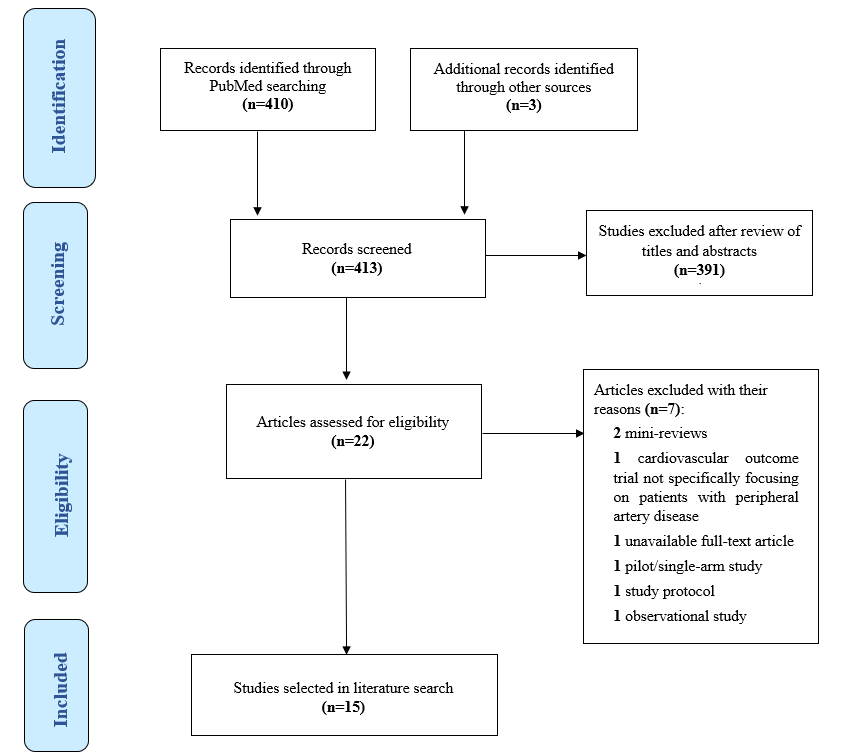


**Fig. S1** Flow diagram of the selection process of the cardiovascular outcome trials including patients with peripheral artery disease.
